# The association between oral health clusters and oral health‐related quality of life: A population‑based study of 5‑yr‑old children

**DOI:** 10.1111/eos.70100

**Published:** 2026-04-30

**Authors:** Pedro Henrique Duarte França de Castro, Maria Augusta Bessa Rebelo, Mario Vianna Vettore

**Affiliations:** ^1^ School of Dentistry Federal University of Amazonas Manaus Amazonas Brazil; ^2^ Department of Dentistry and Oral Health Aarhus University Aarhus Denmark

**Keywords:** child, dental caries, malocclusion, patient reported outcome measures, preschool

## Abstract

This study aimed to describe oral health clusters that combine dental conditions and perceived oral health and to examine the association of oral health indicators and their clusters with oral health‐related quality of life (OHRQoL) in 5‐yr‐old children. This cross‐sectional study analysed data from 4354 5‐yr‐old children participating in the Brazilian National Oral Health Survey (SB Brasil 2023). Oral health assessment included decayed, missing and filled teeth; clinical consequences of untreated dental caries (pufa index); malocclusion; perceived oral health; and OHRQoL (SOHO‐5). Latent profile analysis was performed to identify oral health clusters, and regression models examined associations between clusters, oral health indicators and OHRQoL. Four distinct clusters emerged: healthy teeth and very good/good perceived oral health (56.3%), restored teeth and good/fair perceived oral health (8.9%), high caries and good/fair perceived oral health (24.9%) and high caries/high pufa and fair/poor perceived oral health (9.9%). Oral health indicators (except malocclusion) and oral health clusters were associated with worse OHRQoL, but cluster‐based associations were stronger. Children in clusters with high caries, pufa and poor perceived oral health showed markedly poorer OHRQoL across all domains. This approach may explain OHRQoL better than oral health indicators, providing a more nuanced understanding of oral health disparities.

## INTRODUCTION

Contemporary oral health assessment considering patients’ perspectives along with the use of dental conditions represents a paradigm shift in the evaluation of individual's oral health. The most recent definition of oral health, proposed by the FDI World Dental Federation, considers that functional dimensions, such as speaking, smiling and chewing, together with emotional aspects and the absence of symptoms (e.g., pain and discomfort), are essential components of an individual's oral health [1]. This concept supports the integration of clinical data and patient‐reported outcomes in epidemiological research and oral health programmes [2].

Oral health‐related quality of life (OHRQoL) is a multidimensional construct aligned with the current concept of oral health, encompassing functional, emotional and social impacts of oral health on individual's quality of life [3, 4]. OHRQoL measures evaluate how oral health conditions affect activities such as eating, speaking, sleeping, social interaction and participation in daily routines [5, 6]. Preschool children have limited recall of past experiences, including on the perception of their oral [7, 8]. Therefore, combining the child's self‐perception with guardians’ assessments offers a more comprehensive evaluation of their OHRQoL [9, 10]. Incorporating these measures supports more effective health‐related decision‐making [11].

Evidence from medical literature indicates that clusters of diseases are associated with poorer health‐related quality of life in children [12]. Even though such approach seems equally relevant to dentistry, previous research on multimorbidity of dental conditions in children has primarily reported the relationship between the greater number of dental conditions and poor OHRQoL [13]. Considering the complex care needs of children with multiple dental conditions, investigating oral health clusters and their relationship with OHRQoL warrants further study.

Data‐driven approaches to characterising oral health clusters, particularly those involving children's dental conditions and perceived oral health, are crucial for understanding how oral health problems aggregate at the population level. The inclusion of perceived oral health in the cluster analysis allows the identification of subgroups that reflect not only clinical status but also the subjective experience of oral health, which may capture dimensions not fully explained by clinical indicators alone. This approach provides a more comprehensive characterisation of oral health profiles and consequently to identify targeted populations for interventions. Latent profile analysis (LPA) is a robust person‐centred method that accounts for population heterogeneity and uses statistical modelling to classify individuals into profiles based on shared patterns across continuous, ordinal and count variables while allowing the integration of covariates to enhance profile interpretation [14, 15]. This analytic framework is particularly advantageous for investigating health‐related quality of life impacts across heterogeneous patient populations [16].

Current research on the predictors of oral health on quality of life has focused primarily on individual dental conditions and general health‐related measures, rather than examining whether cluster of dental conditions influences overall health and quality of life [17, 18, 19, 20]. Advancing the understanding of oral health epidemiology requires the assessment of patterns of dental conditions and an exploration of how oral health clusters influence individuals’ quality of life in a dynamic and integrated manner. There is a dearth of evidence assessing whether oral health indicators or oral health clusters exert more impact on children's OHRQoL.

The importance of adopting multidimensional approaches in dental research is undeniable. Understanding these clusters is essential for moving beyond analyses based on individual dental conditions towards integrated epidemiological profiles. The novelty of this study lies in the identification of clusters of oral health indicators, combining dental clinical conditions and perceived oral health, using LPA in 5‐yr‐old children. Another original aspect is the comparison of the relationship between individual oral health measures and OHRQoL, and clusters of oral health indicators and OHRQoL. Identifying subgroups of children with distinct patterns of dental conditions and perceived oral health, as well as and their influence on OHRQoL can inform tailored preventive strategies and targeted interventions, ultimately enhancing oral health and quality of life. Therefore, this study aimed to identify clusters of dental conditions and perceived oral health among 5‐yr‐old children using a data‐driven approach. The associations of oral health indicators and their clusters with OHRQoL were also investigated. Ultimately, we hypothesised that the association between oral health clusters, comprising dental caries, clinical consequences of untreated dental caries, tooth loss and malocclusion, and OHRQoL would be stronger than when oral health indicators were examined as exposures.

## MATERIAL AND METHODS

### Study design and population

This cross‐sectional study utilised secondary data from the Brazilian National Oral Health Survey (SB Brasil 2023) [21] for 5‐yr‐old children who had complete data on dental clinical measures, perceived oral health, sociodemographic characteristics and OHRQoL. The study population comprised residents of permanent private households in the urban areas of Brazil in 2023. The complex sampling process considered the geographic domain divided into 53 strata: the Federal District, capitals and interior municipalities of each federative unit. A stratified cluster sample was randomly selected in a single stage by identifying children of this age group in all households within the selected census tracts. The SB Brasil 2023 was performed in accordance with the Declaration of Helsinki and approved by the National Research Ethics Committee (Opinion No. 4.823.054), following the ethical principles of Resolution 466/12 of National Heralth Council. All patients were informed about possible risks and benefits and all gave written informed consent. Technical reports are publicly available online [22, 23]. This manuscript was prepared in accordance with the STROBE (Strengthening the Reporting of Observational Studies in Epidemiology) guidelines [24].

### Data collection

Data were obtained through structured interviews with guardians and oral examinations of the children. All information was recorded using data entry software on mobile devices. Oral examinations were conducted under natural light using dental mirrors and periodontal probes. Examiners underwent a prior calibration process, and those who achieved a Kappa coefficient of 0.61 or higher [25] for dental clinical measures, relative to the expert‐defined consensus (gold standard), were considered qualified. The methodological procedures adopted in this survey followed the World Health Organization (WHO) recommendations for oral health surveys [2].

### Background factors

Background variables included sociodemographic characteristics, such as sex, race/skin colour, family income and mother's years of education. Sex was classified as male or female. Race/skin colour was categorised as White, Black, brown (pardo), yellow (Asian) or indigenous. Family income was assessed according to the number of Brazilian minimum wages (BMW): <1 BMW, 1–2 BMW and >2 BMW. At the time of the survey, one BMW was equivalent to BRL 1320 (approximately $265 in 2023). Mother's years of education completed with approval were recorded according to the following categories: 0–4, 5–8, 9–11 or ≥12 yr.

### Oral health indicators

Variables used in the LPA included dental clinical indicators and perceived oral health. Dental conditions clinically assessed included caries, missing teeth, filled teeth, clinical consequences of untreated dental caries and malocclusion. The decayed, missing and filled (dmft) index was used to assess the experience of dental caries in the deciduous dentition based on WHO codes and criteria [2]. Each component of the dmft index was analysed separately. Clinical consequences of untreated dental caries, evaluating severe caries and associated pathologies, were assessed using the pufa index, which records the number of teeth with pulp involvement, ulceration, fistula or dentoalveolar abscess [26]. Malocclusion was assessed according to four conditions: posterior crossbite (presence), overbite (reduced, open or deep), overjet (increased, edge‐to‐edge or anterior crossbite) and canine relationship (Class II or III) [27]. Each condition was scored as absent (0) or present (1), and the scores were summed to produce a total malocclusion score ranging from 0 to 4. All dental clinical measures were analysed as continuous variables. Children's perceived oral health was assessed using the following question responded to by their guardians: ‘Overall, how would you rate your child's oral health (teeth and gums)?’. Responses were recorded on a five‐point Likert scale: very good, good, fair, poor or very poor.

### Oral health‐related quality of life

Outcomes were based on OHRQoL, evaluated using the validated version of the Self‐Reported Scale of Oral Health Outcomes (SOHO‐5) for the Brazilian population [28, 29]. SOHO‐5 was completed by children and their guardians. It comprises seven items, which children answer using a three‐point Likert scale with illustrative images, whereas guardians respond using a five‐point Likert scale. The total score is calculated by summing all responses for each participant. Cronbach's alpha for SOHO‐5 in the studied sample was 0.835 for children and 0.850 for guardians, indicating very good internal consistency [30].

### Statistical analysis

Sociodemographic characteristics, dental clinical measures, perceived oral health and SOHO‐5 total scores were summarised using frequencies, means and 95% confidence intervals (CIs) for excluded participants (due to missing data) and the analysed sample.

LPA was then conducted to identify underlying clusters of dental conditions and perceived oral health, using generalised structural equation modelling. Indicators were modelled according to their distributional properties, with count variables specified using Poisson distributions and perceived oral health treated as an ordinal outcome using an ordinal logit link. LPA treats class membership as an unobserved categorical variable, assigning individuals to latent profiles with a given probability. The classes were estimated based on six oral health measures: (1) number of decayed teeth, (2) number of missing teeth, (3) number of filled teeth, (4) number of teeth with clinical consequences of untreated dental caries, (5) malocclusion score and (6) perceived oral health. A series of models specifying two to seven clusters was fitted to obtain the best model according to the lowest values of Bayesian Information Criterion (BIC) and Akaike information criterion (AIC) and highest entropy, ensuring both model fit and classification precision. The four‐class model achieved better fit according to AIC and BIC while maintaining an entropy above 0.8, indicating excellent class separation. Therefore, the four‐cluster model was considered the most appropriate (Table S1).

The associations of oral health indicators and their clusters with OHRQoL (SOHO‐5 total score) were examined using Poisson regression to estimate the ratio of mean (RM) and 95% CIs. Ordinal logistic regression was used to estimate odds ratio and 95% CI for SOHO‐5 dimensions, separately for children and guardians. The likelihood‐ratio test of the proportional odds assumption was non‐significant, suggesting that ordinal logistic regression was adequate. The Poisson regression coefficients were multiplied by three on the log scale, indicating a change in the outcome for every three‐unit increase in each oral health indicator. POH was analysed as a three‐category variable: good (very good/good) as reference category, fair and poor (poor/very poor).

Substantial correlations were observed between decayed teeth, pufa index and perceived oral health (Spearman's correlation coefficients >0.5) (Table S2). Similarly, socioeconomic factors, including family income, maternal education and number of goods, were significantly correlated (Table S3). These findings suggest possible collinearity between oral health measures and between socioeconomic variables. Therefore, regression analyses were conducted to test the association between each oral health indicators, oral health clusters and OHRQoL adjusted for sex, race/skin colour, family income and maternal education. The cluster with better oral health status was considered the reference category in the regression analyses.

All analyses were conducted using stata 18.0 (StataCorp LLC), applying sampling weights, primary sampling units and stratification to account for the complex survey design. The sampling weights were constructed using information on the inverse of the probability of selection across all stages of the complex sampling design (municipality, census tract, household and individual), adjusted for non‐response and adjusted through post‐stratification based on sex, age and education level, using data from the 2022 Continuous National Household Sample Survey [23]. The sample selection process, including the number of participants invited, examined and included in the final analytical sample, is detailed in Figure S1. Missing data were addressed using complete‐case analysis, restricting all analyses to participants with complete information on the variables included in this study. Findings from the statistical analysis were deemed significant if *p* < 0.05.

## RESULTS

**FIGURE 1:**
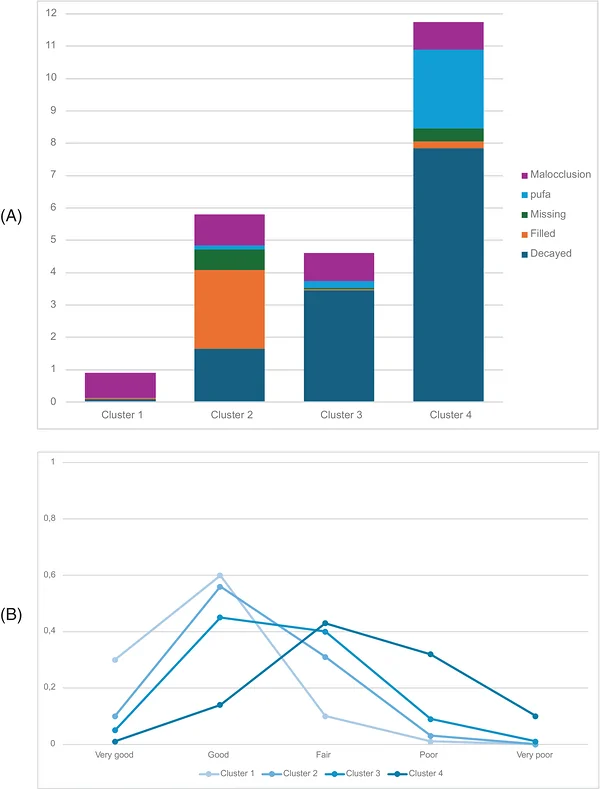
Assessment of latent oral health clusters in 5‐yr‐old children: (A) predicted means of clinical oral health indicators and (B) predicted probabilities of perceived oral health categories.

The final analytic sample consisted of 4354 5‐yr‐old children with complete information for all variables. Descriptive characteristics of participants excluded because of missing data and the analysed sample are presented in Table 1. All variables showed standardised differences below 0.10, indicating that sociodemographic characteristics and oral health measures were similar between the excluded participants and the study sample. Nearly half of the participants were male (49.4%), and 44.8% had brown skin colour. Most children were from families with monthly income of at least two BMWs (63.6%), and 64.8% of the mothers had nine or more years of education (Table 1). The model with four oral health clusters was considered the best LPA solution (Figure 1, Table S4).

**TABLE 1:** Characteristics of the study sample.

	All participants (*n* = 6959)	Participants with missing data (*n* = 2605)	Final analytic sample (*n* = 4354)	
Sociodemographics	% (95% CI)	% (95% CI)	% (95% CI)	SMD
**Sex**				
Male	48.7 (45.8–51.6)	47.5 (41.8–53.2)	49.4 (45.8–53.0)	0.01
Female	51.3 (48.3–54.1)	52.5 (46.8–58.2)	50.6 (47.0–54.2)	0.01
**Skin colour**				
White	43.8 (39.4–48.2)	42.9 (36.0–50.1)	44.3 (39.9–48.7)	0.03
Black	10.4 (8.1–13.1)	12.1 (8.2–17.4)	9.5 (7.2–12.5)	0.09
Yellow	0.8 (0.4–1.3)	0.9 (0.4–1.9)	0.8 (0.4–1.5)	0.06
Brown	44.6 (40.7–48.5)	44.2 (37.9–50.6)	44.8 (40.9–48.8)	0.09
Indigenous	0.4 (0.1–1.6)	0.1 (0.0–0.2)	0.7 (0.2–2.4)	0.05
**Family income (BMW)**				
>3	32.5 (27.5–37.8)	35.7 (27.8–44.4)	32.2 (27.2–37.7)	0.06
2–3	31.6 (27.9–35.4)	34.6 (26.7–43.5)	31.4 (27.6–35.5)	0.02
1–2	13.7 (11.5–16.2)	10.4 (5.4–19.1)	14.0 (11.8–16.5)	0.03
<1	22.2 (17.4–27.8)	19.3 (13.3–27.3)	22.4 (17.6–28.2)	0.08
**Mother's years of education**				
≥12	18.7 (14.3–23.9)	13.7 (9.6–19.3)	20.6 (15.5–26.9)	0.02
9–11	44.3 (40.6–48.1)	44.8 (39.2–50.5)	44.2 (39.4–49.2)	0.01
5–8	8.7 (6.7–11.1)	6.8 (4.7–9.7)	9.5 (7.0–12.8)	0.02
0–4	28.3 (24.4–32.2)	34.7 (27.5–42.8)	25.6 (21.8–29.9)	0.03
**Clinical assessment**	**Mean (95% CI)**	**Mean (95% CI)**	**Mean (95% CI)**	
**dmft**	2.22 (2.15–2.30)	2.17 (2.05–2.29)	2.26 (2.16–2.35)	0.03
Decayed	1.82 (1.75–1.89)	1.75 (1.64–1.85)	1.87 (1.78–1.95)	0.05
Missing	0.10 (0.09–0.12)	0.11 (0.08–0.13)	0.11 (0.09–0.13)	0.01
Filled	0.29 (0.27–0.31)	0.32 (0.28–0.35)	0.28 (0.25–0.30)	0.04
**Pufa**	0.32 (0.29–0.34)	0.31 (0.28–0.35)	0.32 (0.29–0.35)	0.01
**Malocclusion score**	0.81 (0.79–0.84)	0.81 (0.77–0.85)	0.82 (0.79–0.85)	0.01
**Subjective assessment**				
**POH**	**% (95% CI)**	**% (95% CI)**	**% (95% CI)**	
Very good	18.8 (16.4–21.5)	19.3 (16.1–23.1)	18.6 (15.4–22.3)	0.07
Good	50.7 (46.6–54.8)	47.5 (42.2–52.9)	52.5 (47.5–57.3)	0.03
Fair	23.4 (20.5–26.6)	25.6 (20.2–31.8)	22.4 (19.2–25.9)	0.04
Poor	6.0 (4.7–7.5)	7.1 (5.0–10.1)	5.5 (4.1–7.3)	0.01
Very poor	0.9 (0.6–1.4)	0.6 (0.3–1.1)	1.1 (0.7–1.9)	0.05
**SOHO‐5 (by the children)**	**Mean (95% CI)**	**Mean (95% CI)**	**Mean (95% CI)**	
Overall	0.99 (0.75–1.22)	0.72 (0.58–0.85)	1.13 (0.79–1.46)	0.01
Cluster 1	–	–	0.39 (0.26–0.51)	–
Cluster 2	–	–	2.30 (1.11–3.50)	–
Cluster 3	–	–	1.40 (1.00–1.74)	–
Cluster 4	–	–	4.01 (2.61–5.41)	–
**SOHO‐5 (by the child's guardian)**	**Mean (95% CI)**	**Mean (95% CI)**	**Mean (95% CI)**	
Overall	1.25 (0.88–1.61)	0.98 (0.75–1.22)	1.38 (0.87–1.88)	0.03
Cluster 1	–	–	0.46 (0.20–0.73)	–
Cluster 2	–	–	2.97 (1.20–4.75)	–
Cluster 3	–	–	1.41 (1.07–1.89)	–
Cluster 4	–	–	5.44 (3.91–6.98)	–

*Note*: SMD: Standardised difference between participants with missing data and the final analytic sample.

Abbreviation: BMW, Brazilian minimal wage.

The *cluster of healthy teeth and very good/good perceived oral health* (Cluster 1) accounted for 56.3% of the sample and comprised children with lowest mean numbers of decayed, filled and missing teeth; teeth with pufa lesions; and very good/good perceived oral health. The *cluster of restored teeth and good/fair perceived oral health* (Cluster 2) represented 8.9% of the sample and included those with high mean number of filled teeth, moderate levels of decayed teeth, and good/fair perceived oral health. The *cluster of high caries and good/fair perceived oral health* (Cluster 3) comprised 24.9% of the sample and included those with high mean numbers of decayed teeth, low levels of pufa and good/fair perceived oral health. The *cluster of high caries, high pufa and fair/poor perceived oral health* (Cluster 4) represented 9.9% of the sample and encompassed children with highest mean number of decayed teeth and pufa, and fair/poor perceived oral health. Malocclusion was uniformly distributed across clusters, whereas the missing‐teeth component was more concentrated in Clusters 2 and 4. Higher probabilities of worst perceived oral health were consistently clustered with higher levels of untreated dental conditions requiring care.

The adjusted associations between oral health indicators and SOHO‐5 scores are reported in Table 2. Worse oral health measures were consistently associated with higher SOHO‐5 scores. Mean SOHO‐5 scores increased by 70% and 77%, respectively, for each three‐unit increase in the number of decayed teeth when reported by children and their guardians. Children's mean SOHO‐5 scores increased by 2.37, 2.09 and 2.63 points for each three‐unit increase in the number of filled teeth, missing teeth and teeth with clinical consequences of untreated dental caries, respectively. Similarly, a three‐unit increase in the number of filled teeth, missing teeth and teeth with clinical consequences of untreated dental caries was associated with increases of 2.62, 2.57 and 2.97 points, respectively, in guardian's mean SOHO‐5 score. Malocclusion was not associated with SOHO‐5 scores. Children with fair and poor perceived oral health are expected to have a mean of SOHO‐5 scores 5 and 6.7 times higher than those with good perceived oral health. The results were similar when the SOHO‐5 questionnaire was reported by the child's guardian.

**TABLE 2:** Association between oral health indicators and oral health‐related quality of life (OHRQoL) (SOHO‐5) in 5‐yr‐old children.

SOHO‐5	Decayed	Filled	Missing	Pufa	Malocclusion	POH Fair vs. Good	POH Poor vs. Good
	RM/OR (95% CI)	RM/OR (95% CI)	RM/OR (95% CI)	RM/OR (95% CI)	RM/OR (95% CI)	RM/OR (95% CI)	RM/OR (95% CI)
**By the children**							
Total score^a^	**1.70 (1.52–1.90)**	**2.37 (1.73–3.25)**	**2.09 (1.64–2.67)**	**2.63 (2.21–3.12)**	0.94 (0.67–1.31)	**5.04 (4.07–6.25)**	**6.71 (4.90–9.18)**
Difficulty eating^b^	**2.39 (1.99–2.86)**	**3.37 (1.74–6.52)**	**3.40 (1.89–6.12)**	**5.82 (3.25–10.43)**	0.94 (0.55–1.60)	**7.74 (5.40–11.09)**	**19.48 (11.16–34.01)**
Difficulty drinking^b^	**2.05 (1.63–2.57)**	**3.17 (1.45–6.93)**	**2.51 (1.56–4.03)**	**4.93 (2.55–9.55)**	0.80 (0.46–1.38)	**8.08 (5.07–12.89)**	**9.06 (4.96–16.52)**
Difficulty speaking^b^	**1.78 (1.22–2.59)**	**3.24 (1.52–6.88)**	**1.84 (1.08–3.12)**	**3.54 (1.30–9.65)**	0.64 (0.35–1.15)	**6.17 (3.28–11.60)**	**3.88 (2.02–7.45)**
Difficulty playing^b^	**2.09 (1.63–2.68)**	**4.21 (1.98–8.95)**	**3.75 (2.17–6.48)**	**6.17 (3.37–11.29)**	0.92 (0.49–1.74)	**6.59 (3.65–11.90)**	**9.52 (4.43–20.45)**
Avoiding smiling (due to pain)^b^	**2.10 (1.71–2.57)**	**3.23 (1.63–6.37)**	**3.11 (1.79–5.40)**	**5.41 (2.64–11.09)**	0.67 (0.38–1.17)	**7.27 (4.49–11.77)**	**17.63 (10.50–29.62)**
Avoiding smiling (due to appearance)^b^	**1.93 (1.41–2.63)**	**3.25 (1.56–6.79)**	**2.27 (1.41–3.65)**	**5.39 (2.66–10.91)**	1.41 (0.53–3.76)	**7.79 (4.24–14.32)**	**6.91 (3.28–14.53)**
Difficulty sleeping^b^	**2.12 (1.60–2.79)**	**3.46 (1.69–7.05)**	**2.61 (1.60–4.24)**	**5.46 (2.78–10.71)**	0.78 (0.37–1.66)	**7.22 (3.95–13.18)**	**14.46 (7.14–29.28)**
**By the child's guardian**							
Total score^a^	**1.77 (1.63–1.91)**	**2.62 (1.99–3.44)**	**2.57 (1.90–3.47)**	**2.97 (2.57–3.42)**	1.19 (0.78–1.83)	**5.61 (4.20–7.49)**	**8.08 (5.03–12.97)**
Difficulty eating^b^	**2.35 (2.01–2.75)**	**3.78 (2.31–6.18)**	**5.36 (2.77–10.38)**	**6.89 (4.37–10.87)**	1.03 (0.61–1.75)	**8.08 (5.63–11.60)**	**18.18 (10.49–31.49)**
Difficulty drinking^b^	**1.69 (1.33–2.14)**	**3.51 (1.64–7.47)**	**2.37 (1.12–5.05)**	**4.26 (2.34–7.77)**	1.50 (0.52–4.28)	**4.31 (2.31–8.02)**	**5.89 (2.44–14.25)**
Difficulty speaking^b^	**2.20 (1.76–2.76)**	**3.46 (1.49–8.02)**	**4.35 (2.58–7.33)**	**7.00 (4.43–11.05)**	1.46 (0.58–3.71)	**11.95 (7.35–19.42)**	**15.11 (8.09–28.22)**
Difficulty playing^b^	**2.41 (2.04–2.84)**	**3.70 (2.04–6.71)**	**5.47 (2.64–11.30)**	**6.03 (3.80–9.55)**	1.15 (0.58–2.26)	**10.14 (6.57–15.65)**	**26.02 (13.69–49.44)**
Avoiding smiling (due to pain)^b^	**2.05 (1.68–2.51)**	**3.35 (1.53–7.33)**	**1.67 (0.90–3.08)**	**6.40 (3.44–11.91)**	1.10 (0.39–3.07)	**7.15 (4.49–11.37)**	**8.97 (4.46–18.05)**
Avoiding smiling (due to appearance)^b^	**2.36 (1.82–3.05)**	**4.29 (2.01–9.13)**	**3.81 (2.05–7.07)**	**6.56 (4.23–10.18)**	0.88 (0.30–2.54)	**15.09 (9.75–23.36)**	**20.41 (10.26–40.61)**
Difficulty sleeping^b^	**2.62 (1.94–3.53)**	**3.67 (1.77–7.63)**	**2.70 (1.52–4.78)**	**8.06 (3.96–16.38)**	0.75 (0.42–1.34)	**12.31 (7.38–20.51)**	**15.55 (7.83–30.85)**

*Note*: All estimates are adjusted for sex, race/skin colour, family income and maternal education. The coefficients express the effect associated with a three‑unit increase in the clinical predictor. Values in bold indicate *p* < 0.05.

Abbreviations: POH, perceived oral health; RM, ratio of mean.

^a^ Poisson regression with robust variance, estimates refer to ratio of mean (RM).

^b^ Ordinal logistic regression, estimates refer to odds ratio (OR).

The results from the regression analyses on the associations between oral health clusters and OHRQoL are shown in Table 3. Children in Cluster 2 (restored teeth and good/fair perceived oral health) had mean SOHO‐5 scores that were 5.74 times higher than those in Cluster 1 (healthy teeth and very good/good perceived oral health). Mean SOHO‐5 scores among children in Cluster 3 (high caries and good/fair perceived oral health) and Cluster 4 (high caries, high pufa and fair/poor perceived oral health) were 3.34 and 9.44 times higher, respectively, compared with those in Cluster 1. More adverse oral health profiles, particularly those characterised by untreated disease and worse perceived oral health, were strongly associated with higher SOHO‐5 scores. These associations were also observed when OHRQoL was reported by the child's guardian.

**TABLE 3:** Association between oral health clusters and oral health‐related quality of life (OHRQoL) (SOHO‐5) in 5‐yr‐old children.

	Cluster 1 Healthy teeth and very good/good POH (*n* = 2451)	Cluster 2 Restored teeth and good/fair POH (*n* = 388)	Cluster 3 High caries and good/fair POH (*n* = 1082)	Cluster 4 High caries, high pufa and fair/poor POH (*n* = 433)
SOHO‐5	Ref.	RM/OR (95% CI)	RM/OR (95% CI)	RM/OR (95% CI)
**By the children**				
Total score^a^	1.0	**5.74 (3.89–8.47)**	**3.34 (2.37–4.69)**	**9.44 (6.43–13.86)**
Difficulty eating^b^	1.0	**9.39 (5.35–16.47)**	**5.96 (3.89–9.14)**	**24.98 (14.11–44.24)**
Difficulty drinking^b^	1.0	**9.87 (5.08–19.15)**	**4.75 (2.66–8.46)**	**17.94 (8.72–36.89)**
Difficulty speaking^b^	1.0	**5.25 (2.43–11.34)**	**2.70 (1.52–4.80)**	**8.82 (2.18–35.72)**
Difficulty playing^b^	1.0	**9.55 (4.43–20.60)**	**3.05 (1.47–6.36)**	**17.78 (8.21–38.49)**
Avoiding smiling (due to pain)^b^	1.0	**10.29 (4.80–22.07)**	**3.92 (2.28–6.74)**	**19.31 (9.24–40.36)**
Avoiding smiling (due to appearance)^b^	1.0	**3.94 (1.69–9.21)**	1.98 (0.88–4.45)	**12.21 (4.22–35.31)**
Difficulty sleeping^b^	1.0	**5.43 (2.53–11.68)**	**2.90 (1.43–5.91)**	**17.18 (6.50–45.42)**
**By the child's guardian**				
Total score^a^	1.0	**5.92 (4.24–8.27)**	**2.85 (1.70–4.81)**	**10.32 (7.51–14.19)**
Difficulty eating^b^	1.0	**10.91 (6.83–17.45)**	**5.26 (3.68–7.52)**	**24.48 (15.57–38.49)**
Difficulty drinking^b^	1.0	**5.69 (2.96–10.96)**	2.47 (0.98–6.22)	**7.73 (3.28–18.20)**
Difficulty speaking^b^	1.0	**6.93 (3.65–13.13)**	**3.82 (1.57–9.34)**	**25.96 (13.21–50.99)**
Difficulty playing^b^	1.0	**16.06 (8.50–30.33)**	**5.12 (2.85–9.17)**	**29.63 (15.94–55.10)**
Avoiding smiling (due to pain)^b^	1.0	**2.98 (1.50–5.93)**	1.68 (0.73–3.85)	**11.00 (5.48–22.07)**
Avoiding smiling (due to appearance)^b^	1.0	**9.71 (4.99–18.89)**	**3.88 (1.43–10.49)**	**25.42 (11.60–55.69)**
Difficulty sleeping^b^	1.0	**8.61 (4.57–16.23)**	**3.51 (1.78–6.93)**	**32.68 (14.63–73.00)**

*Note*: All estimates are adjusted for sex, race/skin colour, family income and maternal education. Values in bold indicate *p* < 0.05.

Abbreviations: POH, perceived oral health; RM, ratio of mean.

^a^ Poisson regression with robust variance, estimates refer to ratio of mean (RM).

^b^ Ordinal logistic regression, estimates refer to odds ratio (OR).

Overall, children in Clusters 2, 3 and 4 showed higher odds of greater SOHO‐5 domains than those in Cluster 1 (Table 3). The odds of poorer SOHO‐5 domains were more pronounced among children in Cluster 4, which was observed when the outcomes were informed by children and their guardians. The strongest associations when SOHO‐5 was reported by the child and their guardians were, respectively, between Cluster 4 and difficulty eating (OR = 24.98) and between Cluster 4 and difficulty sleeping (OR = 32.68).

## DISCUSSION

This study investigated associations between oral health indicators, oral health clusters and OHRQoL among 5‐yr‐old children. Robust and consistent associations were observed for both approaches in relation to SOHO‐5 impacts. However, malocclusion was not associated with SOHO‐5. These findings were consistent regardless of whether SOHO‐5 was reported by the child or by the guardians.

Four distinct oral health clusters among 5‐yr‐old children were identified when dental clinical measures were integrated with guardian‐proxy perceptions of their child's oral health. The identification of complex clusters involving oral health measures represents a shift from the use of traditional single‐disease metrics. More than half of the children exhibited a healthy pattern. Meanwhile, the other three oral health clusters comprised children with distinct profiles, characterised predominantly by the presence of restored teeth, high levels of caries, and, lastly, a combination of high levels of caries and clinical consequences of untreated dental caries.

Dental conditions and perceived oral health were coherently clustered at the population level in 5‐yr‐old children. Previous investigations have examined the relationship between perceived oral health and dental clinical status, but these studies focused on oral health indicators, included other age groups, used small samples and did not use representative and probabilistic samples [31, 32, 33]. Parental perceptions of their child's oral health are predominantly influenced by dental conditions that present symptoms, such as dental caries and toothache [31, 32, 33]. In contrast, other dental conditions, such as malocclusion, usually show a weaker association with guardian's perceptions of their child's oral health [32, 34]. This finding may be partly explained by the age group studied, as 5‐yr‐old children are less likely to experience or express oral impacts related to dental aesthetics. Consequently, malocclusion may have a limited influence on OHRQoL in early childhood [34]. The present findings underscore the value of a cluster‐based approach, demonstrating that the integration between clinical and subjective oral health measures offers a more comprehensive understanding of children's oral health profiles at the population level, which is relevant for identifying high‐need groups for oral health care.

The assessment of OHRQoL using the SOHO‐5 instrument demonstrated adequate internal consistency in the studied sample. In addition, the findings on the associations of oral health indicators and their clusters with OHRQoL total and domain scores were complementary when the children and their guardians completed the SOHO‐5. These findings suggest that SOHO‐5 was a valid and reliable measure of OHRQoL and align with previous evidence indicating that 5‐yr‐old children are capable of reporting their own perceptions of oral impacts [28]. However, higher means ratios and odds ratios were observed when SOHO‐5 was completed by the guardians. This indicates that symptoms and emotional aspects related to oral health captured by SOHO‐5 reflect experiences accumulated over the child's lifetime, which is less precisely identified by children than by their parents. In addition, guardian proxy reports of children's OHRQoL may be influenced by differences in perception, expectations or awareness of the child's dental status, potentially leading to overestimation or differential reporting of oral impacts. The stronger associations observed in guardian reports may be partly explained by the combination of broader recall of the child's oral health experiences and parental proxy‐reporting biases.

The clusters characterised by a higher burden of dental disease were more strongly associated with OHRQoL. Notably, the magnitude and robustness of the associations observed for oral health clusters were substantially greater than those identified when oral health indicators were analysed individually, including in studies that examined dental caries in isolation [20]. However, these differences should be interpreted with caution, as the measurements of dental conditions (e.g., dental indices and type of variables) and analytical methods used to examine clusters and individual conditions differed and may partly account for the observed discrepancies [35]. Nevertheless, these findings suggest that combining clinical and subjective oral health measures is more sensitive for capturing the complexity of children's oral health, recognising its interplay with OHRQoL, and highlighting its relevance for informing targeted oral health interventions.

The use of LPA represents an important strength of this study as it allowed the identification of clinically and subjectively meaningful oral health patterns [14, 15, 16]. The incorporation of perceived oral health provided insights into functional and psychosocial dimensions relevant to children's quality of life. Additionally, the use of a probabilistic, nationally representative sample enhances the generalisability of the findings and supports robust characterisation of oral health profiles in 5‐yr‐old children.

The following limitations regarding the validity and interpretation of this study should be acknowledged. A substantial number of participants were excluded due to missing data. However, selection bias was unlikely since excluded and included participants did not differ in oral health or socioeconomic characteristics. Involving young children in questionnaire‐based research may raise concerns about recall bias, particularly when they are asked to complete the SOHO‐5 instrument to report their own experiences. The inclusion of guardian‐reported assessments of their children's OHRQoL was intended to mitigate this limitation by providing complementary information [7, 9, 10]. The interpretation of the associations between number of filled teeth, the oral health cluster characterised by restored teeth (Cluster 2) and OHRQoL should be approached with caution due to cross‐sectional study design. According to SOHO‐5, the evaluation of OHRQoL is based on the occurrence of oral impacts during the child's lifetime. Therefore, it is difficult to disentangle the temporal relationship between dental conditions and OHRQoL when previous dental problems have already been treated. It can be assumed that some children with restored teeth may have reported oral impacts due to past caries that have been treated, which no longer reflect their current oral health status, potentially overestimating the association between restored teeth and OHRQoL. The potential shared‐method variance due to assessing perceived oral health and OHRQoL using self‐reported measures should be considered when interpreting the findings. Perceived oral health was a meaningful indicator to model oral health clusters. However, the use of self‐reported instruments for both exposure and outcome may artificially overestimate the observed associations due to common‐method bias.

The reported association between oral health clusters and OHRQoL has potential implications for policy and practice. These findings suggest that using multidimensional oral health indicators can improve the performance of health surveillance systems, support resource allocation, and inform the development of targeted preventive and therapeutic strategies. The cross‐sectional design should be considered when interpreting the present findings. Future longitudinal studies are needed to characterise the trajectories of oral health clusters over time and to determine patterns of oral health clusters in other age groups.

In conclusion, the shift from analysing oral health measures separately to adopting an integrated, profile‐based approach may offer a complementary perspective, representing a significant advancement in oral epidemiology. Our findings indicate that distinct oral health clusters are associated with OHRQoL. The present evidence may contribute to informing policy development and planning aimed at reducing oral health inequalities.

## AUTHOR CONTRIBUTIONS

**Conceptualisation**: Pedro Henrique Duarte França de Castro, Maria Augusta Bessa Rebelo, and Mario Vianna Vettore. **Methodology**: Pedro Henrique Duarte França de Castro, Maria Augusta Bessa Rebelo, and Mario Vianna Vettore. **Formal analysis**: Pedro Henrique Duarte França de Castro, Maria Augusta Bessa Rebelo, and Mario Vianna Vettore. **Data curation**: Pedro Henrique Duarte França de Castro and Mario Vianna Vettore. **Funding acquisition**: Pedro Henrique Duarte França de Castro and Maria Augusta Bessa Rebelo. **Writing—original draft preparation**: Pedro Henrique Duarte França de Castro, Maria Augusta Bessa Rebelo, and Mario Vianna Vettore. **Writing—review and editing**: Pedro Henrique Duarte França de Castro, Maria Augusta Bessa Rebelo, and Mario Vianna Vettore.

## CONFLICT OF INTEREST STATEMENT

The authors declare no conflicts of interest.

## ETHICS STATEMENT

The Brazilian National Oral Health Survey (SB Brasil 2023) was performed in accordance with the Declaration of Helsinki 1975, as revised in 2008, and approved by the National Research Ethics Committee (Opinion no. 4.823.054), following the ethical principles of Resolution 466/12 of the National Health Council. All patients were informed about possible risks and benefits, and all gave written informed consent.

## Supporting information



Supporting Information
