# Supplementary material for: The association between oral health clusters and oral health‐related quality of life: A population‑based study of 5‑yr‑old children
Source: Eur J Oral Sci. 2026 Apr 30;134(5):e70100. doi: 10.1111/eos.70100 (PMC13584666; doi:10.1111/eos.70100)
Supplement: Supplementary file 1 [file EOS-134-e70100-s001.docx]

**The association between oral health clusters and oral health-related quality of life: a population‑based study of five‑year‑old children**

Pedro Henrique Duarte França de Castro^1,2^, Maria Augusta Bessa Rebelo^1^, Mario Vianna Vettore^2^

^1^ School of Dentistry, Federal University of Amazonas, Manaus, Amazonas, Brazil

^2^ Department of Dentistry and Oral Health, Aarhus University, Aarhus C, Denmark

**Supporting Information**

| **Table S1** - Model fit statistics for Latent Profile Analysis (LPA). | | | | | |
| --- | --- | --- | --- | --- | --- |
| **Model** | **Log-Likelihood** | **df** | **AIC** | **BIC** | **Entropy** |
| 2 Clusters | -25,408.80 | 10 | 50,855.60 | 50,976.80 | 0.894 |
| 3 Clusters | -24,325.23 | 10 | 48,708.47 | 48,893.46 | 0.876 |
| 4 Clusters | -23,597.42 | 10 | 47,272.84 | 47,521.62 | 0.863 |
| 5 Clusters | **-** | **-** | **-** | **-** | - |
| 6 Clusters | **-** | **-** | **-** | **-** | **-** |
| 7 Clusters | **-** | **-** | **-** | **-** | **-** |
| - Convergence not achieved for models with 5 to 7 clusters. | | | | | |

| **Table S2 -** Spearman correlation coefficients between dental conditions and perceived oral health. | | | | | | |
| --- | --- | --- | --- | --- | --- | --- |
|  | **Decayed** | **Filled** | **Missing** | **pufa** | **Malocclusion** | **Perceived oral health** |
| **Decayed** | 1.0000 |  |  |  |  |  |
| **Filled** | 0.0966* | 1.0000 |  |  |  |  |
| **Missing** | 0.2145* | 0.1554* | 1.0000 |  |  |  |
| **pufa** | 0.5271* | 0.0358 | 0.1610* | 1.0000 |  |  |
| **Malocclusion** | 0.0293 | 0.0398 | -0.0003 | 0.0211 | 1.0000 |  |
| **Perceived oral health** | 0.5223* | 0.0921* | 0.1351* | 0.3707* | 0.0184 | 1.0000 |
| * p<0.001 | | | | | | |

| **Table S3 -** Spearman correlation coefficients between socioeconomic variables. | | | | |
| --- | --- | --- | --- | --- |
|  | **Family income** | **Household crowding** | **Number of durable goods** | **Mother’s years of education** |
| **Family income** | 1.000 |  |  |  |
| **Household crowding** | -0.254* | 1.000 |  |  |
| **Number of durable goods** | 0.601* | -0.253* | 1.000 |  |
| **Mother’s years of education** | 0.357* | -0.135* | 0.321* | 1.000 |
| * p<0.001 |  |  |  |  |

| **Table S4 -** Marginal means of oral health variables across oral health clusters identified by Latent Profile Analysis (LPA). | | | | | | | |
| --- | --- | --- | --- | --- | --- | --- | --- |
| **Cluster** | **Variable** | **Margin** | **Std. Err.** | **z** | **95% CI**  **Lower** | **95% CI**  **Upper** |  |
| 1 | Decayed | 0.09 | 0.01 | 7.63 | 0.07 | 0.11 |  |
|  | Filled | 0.03 | 0.01 | 4.88 | 0.02 | 0.05 |  |
|  | Missing | 0.00 | 0.00 | 2.42 | 0.00 | 0.01 |  |
|  | pufa | 0.00 | 0.00 | 15.45 | 0.00 | 0.00 |  |
|  | Malocclusion | 0.78 | 0.02 | 35.65 | 0.73 | 0.82 |  |
|  | Perceived oral health |  |  |  |  |  |  |
|  | Very good | 0.30 | 0.01 | - | 0.28 | 0.32 |  |
|  | Good | 0.60 | 0.01 | - | 0.58 | 0.62 |  |
|  | Fair | 0.10 | 0.01 | - | 0.09 | 0.11 |  |
|  | Poor | 0.01 | 0.00 | - | 0.00 | 0.01 |  |
|  | Very poor | 0.00 | 0.00 | - | 0.00 | 0.00 |  |
| 2 | Decayed | 1.64 | 0.16 | 10.57 | 1.34 | 1.94 |  |
|  | Filled | 2.44 | 0.13 | 18.75 | 2.19 | 2.70 |  |
|  | Missing | 0.63 | 0.08 | 7.49 | 0.47 | 0.80 |  |
|  | pufa | 0.14 | 0.03 | 4.86 | 0.09 | 0.20 |  |
|  | Malocclusion | 0.95 | 0.06 | 14.57 | 0.82 | 1.07 |  |
|  | Perceived oral health |  |  |  |  |  |  |
|  | Very good | 0.10 | 0.02 | - | 0.07 | 0.13 |  |
|  | Good | 0.56 | 0.03 | - | 0.50 | 0.61 |  |
|  | Fair | 0.31 | 0.03 | - | 0.26 | 0.36 |  |
|  | Poor | 0.03 | 0.01 | - | 0.01 | 0.07 |  |
|  | Very poor | 0.00 | 0.00 | - | 0.00 | 0.03 |  |
| 3 | Decayed | 3.45 | 0.16 | 21.88 | 3.14 | 3.75 |  |
|  | Filled | 0.04 | 0.01 | 3.02 | 0.01 | 0.07 |  |
|  | Missing | 0.04 | 0.01 | 3.43 | 0.02 | 0.07 |  |
|  | pufa | 0.21 | 0.03 | 7.59 | 0.16 | 0.27 |  |
|  | Malocclusion | 0.87 | 0.04 | 22.29 | 0.79 | 0.94 |  |
|  | Perceived oral health |  |  |  |  |  |  |
|  | Very good | 0.05 | 0.01 | - | 0.04 | 0.07 |  |
|  | Good | 0.45 | 0.02 | - | 0.41 | 0.49 |  |
|  | Fair | 0.40 | 0.02 | - | 0.37 | 0.44 |  |
|  | Poor | 0.09 | 0.01 | - | 0.07 | 0.12 |  |
|  | Very poor | 0.01 | 0.00 | - | 0.00 | 0.02 |  |
| 4 | Decayed | 7.85 | 0.22 | 36.44 | 7.43 | 8.27 |  |
|  | Filled | 0.21 | 0.05 | 4.39 | 0.12 | 0.31 |  |
|  | Missing | 0.39 | 0.07 | 5.56 | 0.25 | 0.53 |  |
|  | pufa | 2.45 | 0.19 | 12.76 | 2.08 | 2.83 |  |
|  | Malocclusion | 0.84 | 0.06 | 14.22 | 0.72 | 0.95 |  |
|  | Perceived oral health |  |  |  |  |  |  |
|  | Very good | 0.01 | 0.01 | - | 0.00 | 0.03 |  |
|  | Good | 0.14 | 0.02 | - | 0.1 | 0.18 |  |
|  | Fair | 0.43 | 0.03 | - | 0.38 | 0.49 |  |
|  | Poor | 0.32 | 0.02 | - | 0.27 | 0.37 |  |
|  | Very poor | 0.1 | 0.02 | - | 0.07 | 0.14 |  |

**Figure S1** Flowchart of the sample selection process for the study.
